# Multifocal Epidural Neurosarcoidosis Causing Spinal Cord Compression: A Case Report

**DOI:** 10.7759/cureus.4177

**Published:** 2019-03-04

**Authors:** Michael Longo, Yaroslav Gelfand, Merritt D Kinon, James Pullman, Reza Yassari

**Affiliations:** 1 Neurosurgery, Albert Einstein College of Medicine, Montefiore Medical Center, Bronx, USA

**Keywords:** neurosarcoidosis, epidural, management

## Abstract

We describe a rare case of multifocal extramedullary epidural neurosarcoidosis that presented with myelopathy without motor deficits and perform a literature review for previous cases of epidural neurosarcoidosis. A 46-year-old woman presented with lower back pain, urinary incontinence, gait disturbance, and sensory loss without motor deficits. Spine magnetic resonance imaging (MRI) showed multiple epidural lesions, the largest causing spinal cord compression at the T5 level. A computed tomography (CT)-guided biopsy of the dominant lesion showed noncaseating granulomas consistent with neurosarcoidosis. She was treated with a course of dexamethasone and discharged home after a 10-day hospital course. She was discharged home on oral prednisone taper over a four-month period. At her latest follow-up, she is neurologically intact and gainfully employed. This case demonstrates that certain cases of epidural neurosarcoidosis causing spinal cord compression may be treated with medical therapy alone in the absence of severe neurological deficits.

## Introduction

Sarcoidosis is a highly mimetic disease process that can affect numerous organ systems. Although the classical association is with pulmonary disease, sarcoid may also affect the central nervous system in approximately 10% of cases. If the spinal cord is affected, the disease is typically intradural and standard management consists of corticosteroids and, in some cases, surgical decompression. Here we present a rare case of multifocal extradural neurosarcoidosis causing myelopathy with spinal cord compression. Her case was successfully managed with medical therapy alone and she achieved full recovery.

## Case presentation

We present here a case of a 46-year-old woman with a past medical history significant for a positive purified protein derivative (PPD) diagnosed in 1997 who presented to the emergency department complaining of severe low back pain. She reported that, as her pain worsened, she began to have difficulty walking, requiring a cane to ambulate. She also endorsed sensory loss below her knees, urinary incontinence, and chills without fever, however her incontinence was ostensibly related to a gynecological issue. She denied smoking, intravenous drug use, alcohol abuse, or recent travel. Her recent medical history was only significant for a mild cold several weeks ago. She presented to the emergency department for back pain two months prior and was discharged home after lumbar and sacral plain films were negative.

Physical exam on this admission was significant for diminished sensation below the knees in non-dermatomal distribution without a sensory level, full strength throughout the upper and lower extremities, and an unsteady gait, corresponding to a Frankel grade of D. Hoffman’s sign was positive bilaterally and she was diffusely hyperreflexic. Rectal tone was intact and no cranial nerve deficits were noted.

Vital signs were within normal range. Her initial labs revealed an elevated white blood cell count of 11.2 k/uL (normal 4.8-10.8 k/uL) with a left shift and erythrocyte sedimentation rate of 40 mm/h (normal 0-20 mm/h). Her C-reactive protein, basic metabolic panel, and liver function studies were within normal limits.

**Figure 1:**
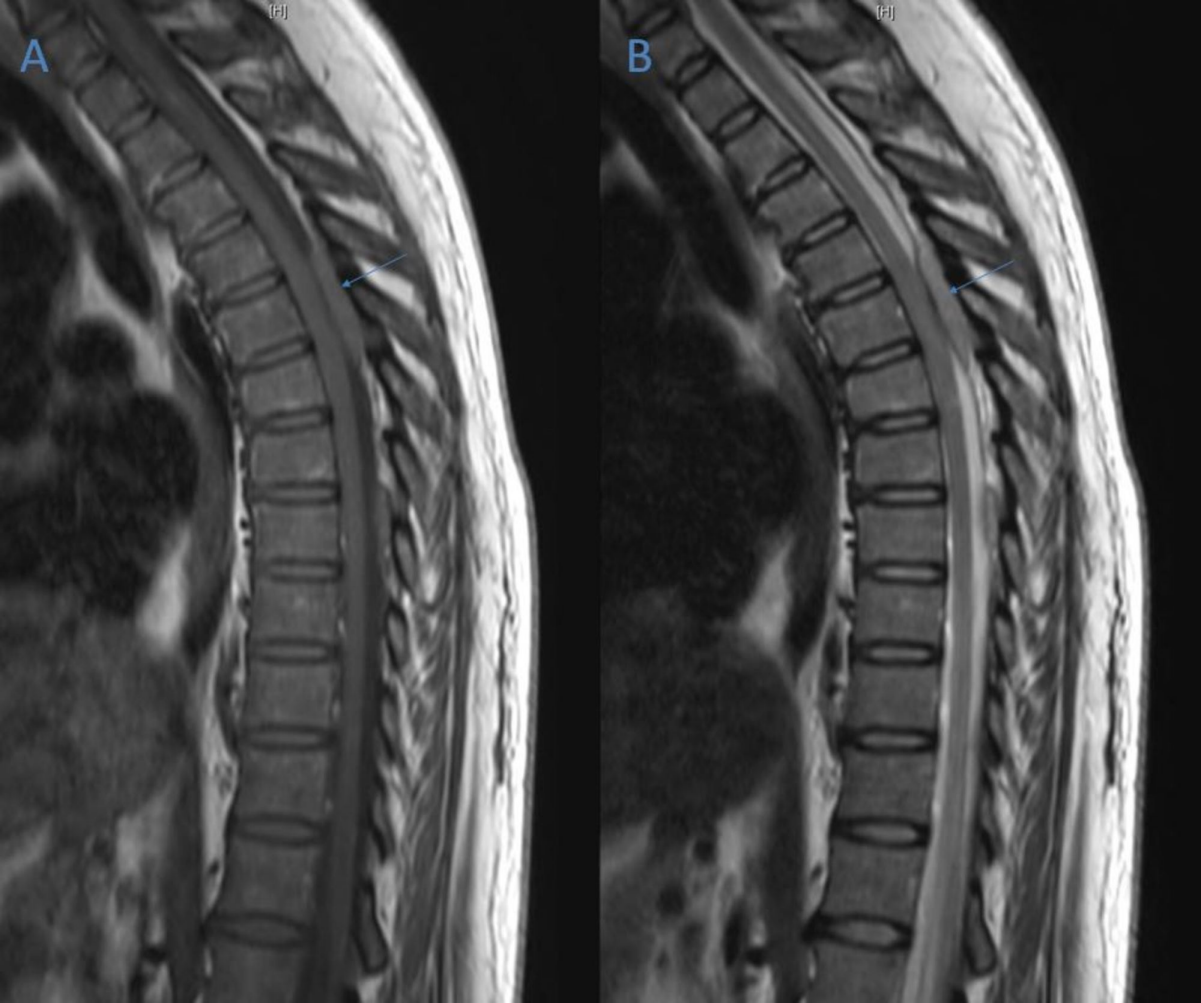
Sagittal Magnetic Resonance Imaging (MRI) from Day One of Admission. (A) Gadolinium-enhanced T1-weighted sagittal MRI showing posterior epidural lesion centered at T5 vertebral level (arrow). (B) Gadolinium-enhanced T2-weighted sagittal MRI showing posterior epidural lesion centered at T5 vertebral level (arrow).

**Figure 2:**
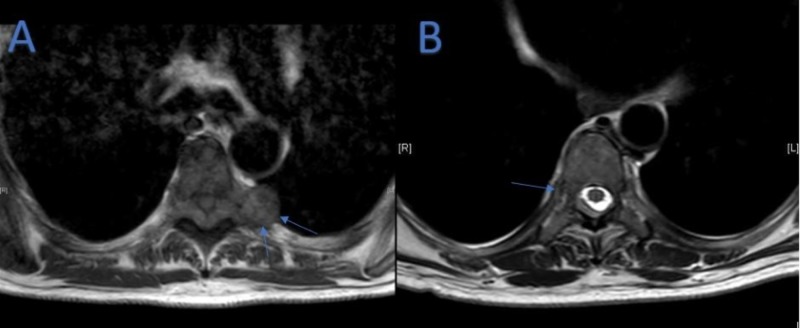
(A) Axial Magnetic Resonance Imaging (MRI) at T5 Vertebral Level on Day One of Admission. (B) Axial MRI at T8 Vertebral Level on Day One of Admission. (A) Gadolinium-enhanced T1-weighted axial MRI on day one of admission showing posterior epidural lesion at T5 vertebral level (arrows). (B) Gadolinium-enhanced T2-weighted axial MRI on day one of admission showing small lesion at T8 vertebral level involving right posterior aspect of T8 vertebral body (arrow).

**Figure 3:**
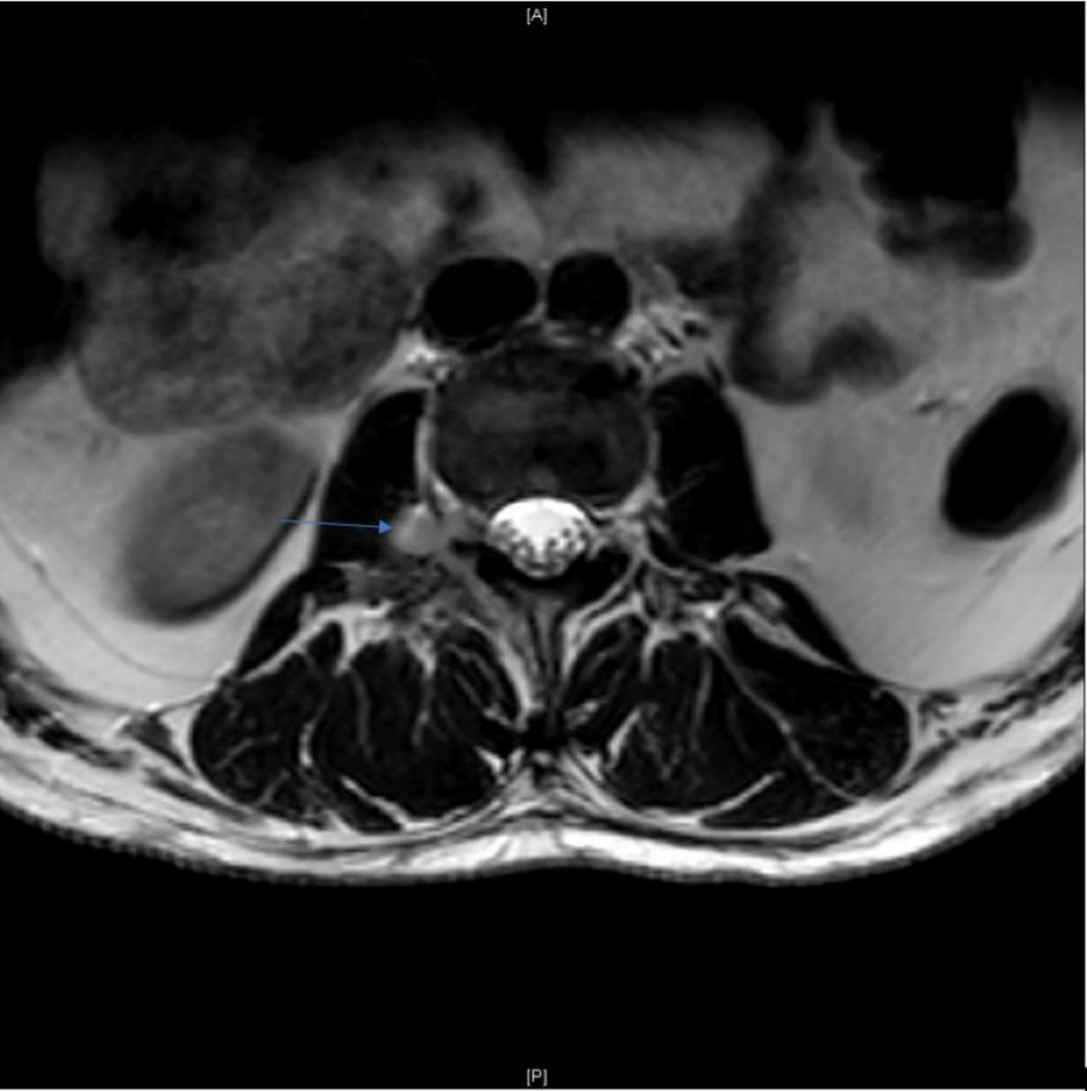
T2W Axial Magnetic Resonance Imaging (MRI) at L2/L3 Level on Day One of Admission. Gadolinium-enhanced T2-weighted axial MRI at the L2/L3 level showing epidural lesion in the right neural foramen (arrow) on day one of admission.

Full spine magnetic resonance imaging (MRI) was performed that revealed three epidural lesions distributed throughout the thoracic and lumbar spine. The largest lesion was centered at the T5 vertebral body and extended from T4-T6 causing spinal cord compression with T2 signal changes (Figure 1A-1B). The lesion involved the vertebral body and was mostly T2 hypointense with contrast enhancement (Figure 2A). An additional T2 hypointense extradural lesion with enhancement involved the right posterior aspect of the T8 vertebral body (Figure 2B). The lumbar lesion was located at the L2-L3 level and extended into the right neural foramen, L2 lamina, and L2 posterior elements (Figure 3). This lesion was also T2 hypointense and demonstrated contrast enhancement. At this point, the differential diagnosis was broad and included neurosarcoidosis, lymphoma, leukemia, epidural abscess, metastatic disease, and disseminated tuberculosis. She was administered a bolus of dexamethasone 10 mg due to spinal cord compression and started on dexamethasone 4 mg every six hours and broad-spectrum antibiotics. Her intact motor function in the presence of sensory disturbances (Frankel grade D), spinal stability, and disease course permitted close monitoring before her case was presented at tumor board, where the decision was made to proceed with a computed tomography (CT)-guided biopsy of the lesion at the T5 level to establish a definitive diagnosis.

**Figure 4:**
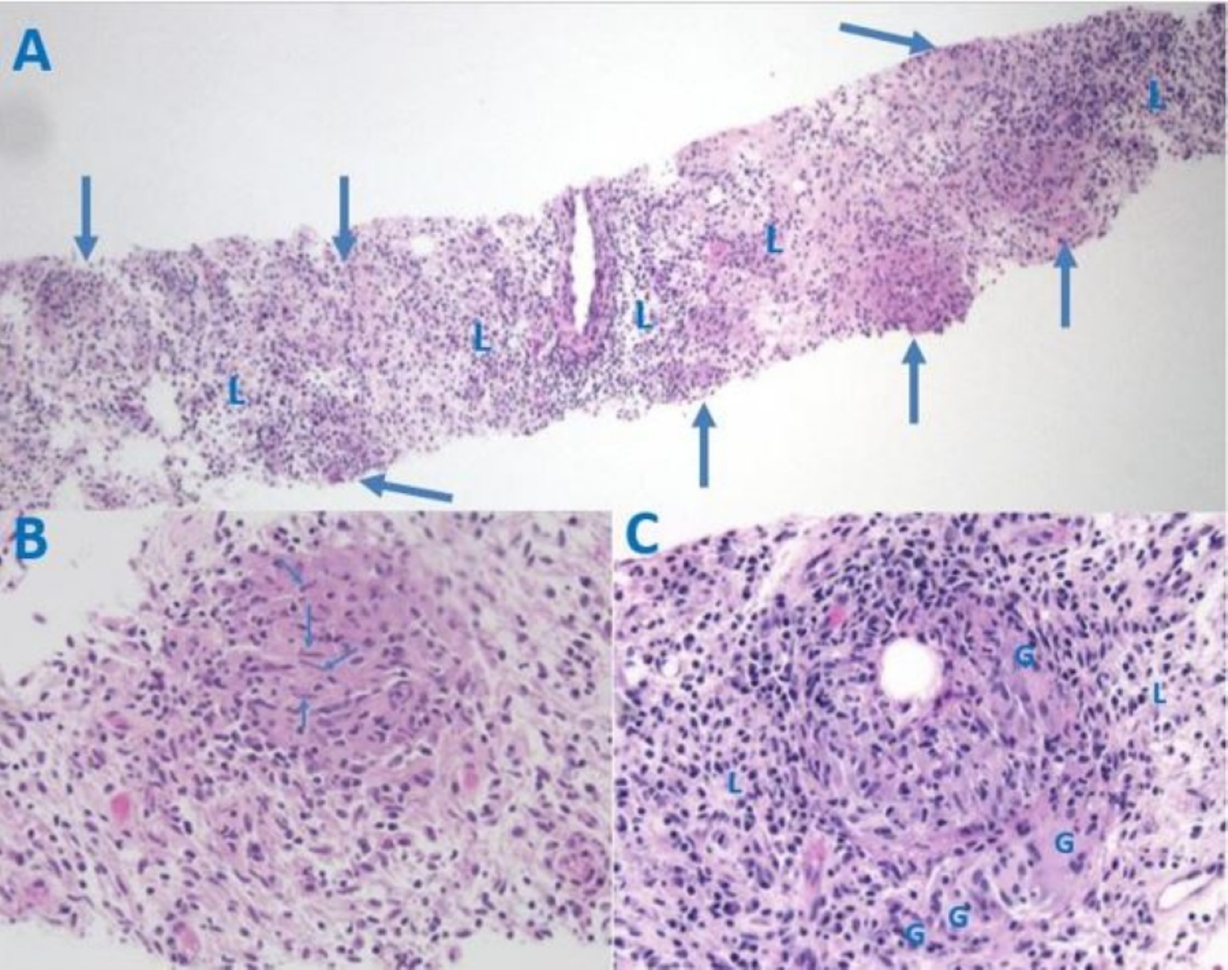
Histology of the Paraspinal Mass, Hematoxylin and Eosin (H&E) Staining. (A) Biopsy core showing non-caseating granulomas (arrows) and lymphocytes in the intervening space (L), 2x original magnification. (B) Enlarged view of one granuloma containing multiple histiocytes characterized by elongated nuclei (arrows), 20x original magnification. (C) Enlarged view of another granuloma with multinucleated giant cells (G) and lymphocytes at the periphery, (L) 20x original magnification.

CT-guided needle-core biopsy of the dominant lesion revealed noncaseating granulomatous inflammation without evidence of fungal or bacterial organisms, including acid fast bacteria, by gram, Fite, acid-fast bacilli (AFB) and Grocott-Methenamine Silver (GMS) stains, or of lymphoma or other neoplasms. Hematoxylin and eosin (H&E) stained sections show the granulomas as multiple nodules at low magnification (Figure 4A), composed of mononuclear histiocytes and multinucleate giant cells seen at higher magnification (Figure 4B-4C). Plasma cells and lymphocytes occupy the space around and between granulomas (Figure 4C). These findings are all consistent with a diagnosis of sarcoidosis. Posterior-anterior and lateral chest films performed for further evaluation of sarcoid were negative: the lungs were clear and the hilar and mediastinal areas were unremarkable, showing no signs of sarcoid-related adenopathy. Also, serum angiotensin-converting-enzyme (ACE) was within normal limits at 12 U/L (normal 8-52 U/L), although this is often elevated in sarcoidosis. Cerebrospinal fluid (CSF) ACE levels were not obtained due to the strong evidence for neurosarcoidosis from the biopsy.

**Figure 5:**
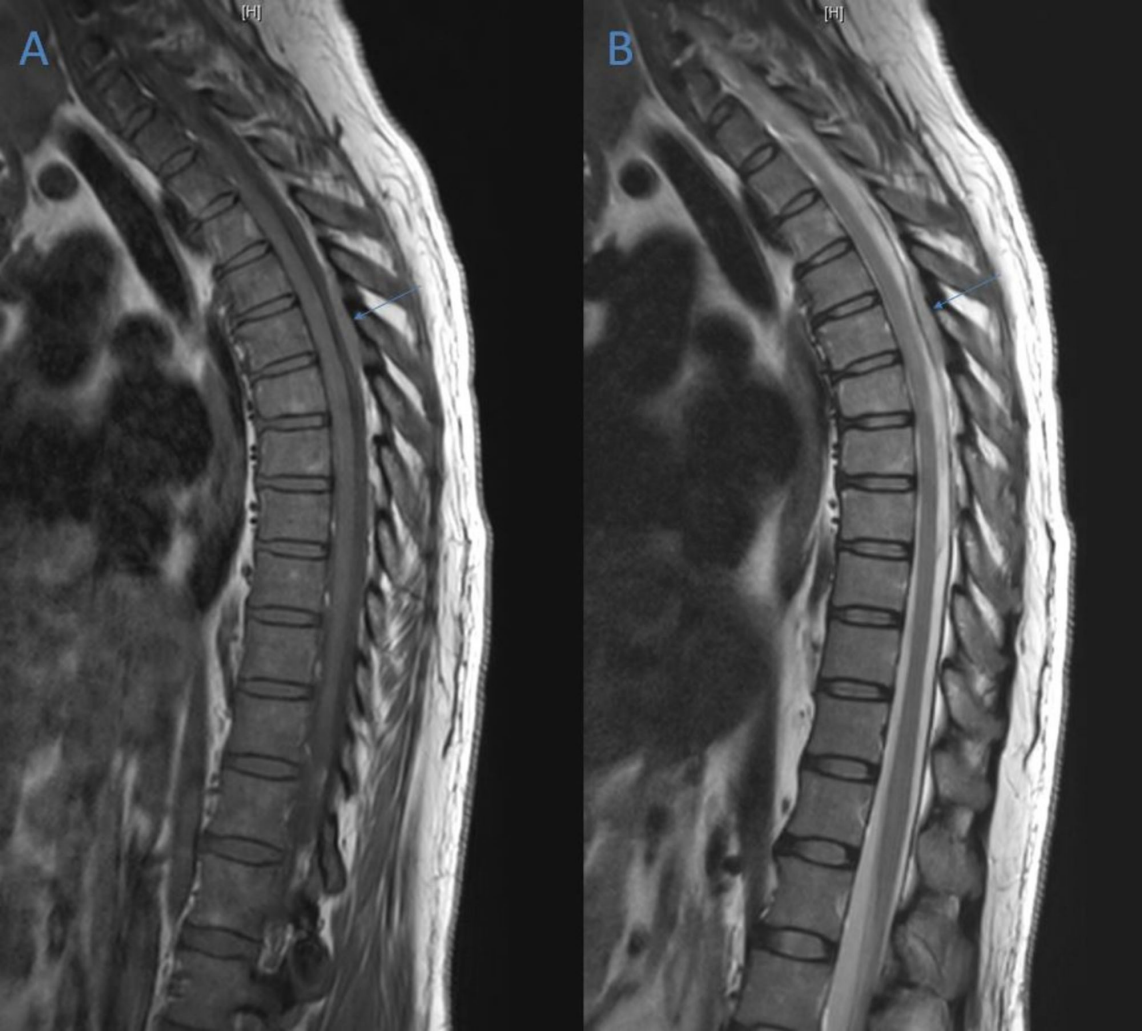
Sagittal Magnetic Resonance Imaging (MRI) from Day Eight of Admission. (A) Gadolinium-enhanced T1-weighted sagittal MRI showing a decrease in size of epidural T5 lesion (arrow). (B) Gadolinium-enhanced T2-weighted sagittal MRI showing a decrease in size of epidural T5 lesion (arrow).

The patient was admitted to the intensive care unit under close neurological monitoring. A follow-up MRI was performed eight days after initial imaging and initiation of corticosteroid therapy to monitor disease response. It showed a significant reduction in the size of the posterior epidural masses centered at T5 and T8 (Figure 5A-5B). Spinal cord edema was also decreased at this level. These factors, coupled with a steady increase in ambulatory stability, made the patient a candidate for discharge to acute rehab. She preferred to go home with services and was discharged on prednisone 80 mg daily on day 10 of admission.

At follow-up the week after discharge, her back pain and gait improved significantly; Karnofsky Performance Status (KPS) was reported as 90. At her latest follow-up over 18 months after initial admission, she has gainful employment, ambulates without difficulty, and is neurologically intact.

## Discussion

Overview of spinal neurosarcoidosis

Sarcoidosis is a common, chronic inflammatory disease of unknown etiology classically defined by noncaseating granulomas on histology [1, 2]. Neurological symptoms occur in 5% to 10% of cases, usually presenting as cranial neuropathy, aseptic meningitis, or cerebral parenchymal disease [1]. Spinal cord involvement occurs in approximately 10% of neurosarcoidosis cases and most often manifests as intramedullary disease (e.g., transverse myelitis) [3-7]. Intradural extramedullary neurosarcoidosis is exceedingly uncommon, and extradural disease is reported even less frequently [3, 7-10]. Diagnostic evidence of spinal neurosarcoidosis may include elevated serum or CSF ACE, hilar lymphadenopathy on chest imaging, increased uptake in implicated regions on Gallium scan, or MRI demonstrating meningeal enhancement [11-13]. However, a definitive diagnosis is principally dependent on a histopathologic specimen that typically shows non-necrotizing granulomas in the setting of chronic inflammation [7, 11, 12]. The specimen is taken from a lymph node or cutaneous lesion in patients with systemic disease, but biopsy of an affected area of the central nervous system is a reasonable approach if there is no evidence of systemic disease and the target lesion is accessible [11].

Although numerous immunosuppressive agents have been used as adjunctive agents in the management of neurosarcoidosis (methotrexate, cyclosporine, azathioprine, infliximab), corticosteroid therapy is the standard for medical care [11, 14]. The regimen typically consists of a six- to eight-week course of oral steroids at 1 mg/kg followed by taper [11]. However, spinal cord disease is frequently more resistant to corticosteroids than cerebral neurosarcoidosis, often requiring a prolonged course. In severe cases which cause focal neurological deficits or spinal instability, surgical decompression may be indicated [11, 14]. Hoitsma et al. describe neurosurgical intervention as indicated only in “life threatening situations or when medical treatment is insufficient” [14].

Cases of epidural neurosarcoidosis

Our review of the literature returned four cases of epidural neurosarcoidosis causing neurological deficits, each presenting as myelopathy without systemic manifestations of sarcoidosis. These confirmed cases of epidural neurosarcoidosis causing spinal cord compression required surgical intervention to forestall the disease [2, 8, 15, 16].

Barazi et al. described a 44-year-old woman who presented with sensory disturbance and mild lower extremity motor deficits whose imaging revealed a T2 hyperintense lesion with contrast enhancement at the thoracolumbar junction [2]. Diagnosis was confirmed by biopsy showing non-necrotizing granulomatous inflammation. She received corticosteroid therapy and underwent a T11-L1 laminectomy. At follow-up the patient was neurologically intact and the disease appeared to be in remission [2]. Nardone et al. described a 52-year-old woman who presented with spastic paraparesis and sensory loss below the T4 level. Imaging revealed a contrast-enhancing mass at T2-T6 circumferentially filling the spinal canal. This patient also had a laminectomy and received corticosteroid therapy, and at follow-up one year later her spine MRI was unremarkable [15]. Weissman et al. described a 37-year-old man who presented with cauda equina syndrome and was found to have a large epidural lesion from L1-S1. He underwent a laminectomy in addition to receiving corticosteroids, and similarly returned to his baseline function. Similarly, diagnosis was confirmed by histopathology showing sarcoid granulomas [16]. The final case in the current literature was of a nine-year-old girl with an epidural lesion reported by Galgano et al. [8]. They describe a patient with recurrent epidural disease requiring multiple surgeries who presented with myelopathy secondary to a thoracic lesion. Disease persistence was presumably due to corticosteroid noncompliance. A presumptive diagnosis of epidural neurosarcoidosis was made due to granulomatous inflammation on biopsy specimens and a significant response to corticosteroids. The authors note that other pathologies cannot, however, be ruled out [8].

Contrast with the present case

Several similarities are apparent between the above-mentioned cases and ours, such as presentation with myelopathic symptoms, no evidence of systemic disease, and an impressive response to treatment. Yet our case is unique in that the patient presented myelopathy secondary to multiple extradural lesions and did not require surgical intervention, despite the presence of spinal cord compression. Our patient’s full strength on presentation in the presence of minor sensory and gait disturbances (Frankel grade D) combined with a swift response to corticosteroid therapy allowed us to manage the case nonoperatively once pathology confirmed the diagnosis of neurosarcoidosis. It is critical to note that the patient’s neurological status remained stable during her admission and began to improve after steroid administration. In a scenario of refractory medical treatment and/or deteriorating neurological function, surgical decompression must be part of the treatment algorithm and hence close monitoring is imperative while the medical management is ongoing. However, our patient steadily improved on medical management, avoiding the need for surgical decompression.

## Conclusions

Future study of epidural neurosarcoidosis is certainly limited by the rarity of the disease, making large prospective study exceedingly difficult. As such, this case suggests that epidural neurosarcoidosis with spinal cord compression and without major neurological deficits may be successfully managed medically to achieve full recovery.
